# Can Chronic Pain Accelerate Chronic Kidney Disease? Is Pain a Disease Modifier Rather than a Symptom?

**DOI:** 10.3390/life16081354

**Published:** 2026-08-18

**Authors:** Nebojsa Brezic, Vladimir Prelevic, Aleksandar Sic, Marko Baralic, Nebojsa Nick Knezevic

**Affiliations:** 1 Department of Anesthesiology, NewYork-Presbyterian Brooklyn Methodist Hospital, Brooklyn, NY 11215, USA; 2 Clinical Centre of Montenegro, Clinic for Nephrology, 81000 Podgorica, Montenegro; vladimir.scopheurope@gmail.com; 3 School of Medicine, University of Montenegro, 81000 Podgorica, Montenegro; 4 Department of Anesthesiology, Advocate Illinois Masonic Medical Center, Chicago, IL 60657, USA; aca.smed01@gmail.com; 5 Faculty of Medicine, University of Belgrade, 11000 Belgrade, Serbia; 6 Nephrology Clinic, University Clinical Center of Serbia, 11000 Belgrade, Serbia; 7 Department of Anesthesiology, University of Illinois, Chicago, IL 60612, USA; 8 Department of Surgery, University of Illinois, Chicago, IL 60612, USA

**Keywords:** neuroimmune activation, endothelial dysfunction, oxidative stress, chronic inflammation, central sensitization

## Abstract

Chronic pain is one of the most common and disabling complications of chronic kidney disease (CKD), yet it is almost exclusively viewed as a consequence of declining kidney function. This perspective overlooks the possibility that chronic pain itself may influence the biological processes driving CKD progression. Chronic pain is being recognized as a systemic disorder characterized by sustained neuroimmune activation, inflammation, oxidative stress, endothelial dysfunction, autonomic imbalance, mitochondrial impairment and maladaptive behavioral changes. These same mechanisms are also central to progressive kidney injury, raising the possibility that persistent pain may reinforce established pathogenic pathways rather than simply accompany them. We propose a conceptual framework in which chronic pain functions as a disease modifier capable of amplifying the biological and clinical processes that contribute to CKD progression. Although this hypothesis does not imply that chronic pain directly causes CKD or that pain management is inherently renoprotective, it provides a biologically plausible rationale for investigating whether pain contributes to disease progression beyond serving as a marker of disease severity. Exploring this possibility may broaden current concepts of CKD progression and identify new directions for mechanistic, translational and clinical research.

## 1. Introduction

Chronic kidney disease (CKD) affects more than 10% of the global population and remains a major contributor to morbidity and premature mortality worldwide [1]. Although the introduction of kidney-protective therapies has slowed disease progression for many patients, CKD continues to be a substantial symptom burden throughout its course [2]. Chronic pain is among the most common and disabling symptoms, affecting nearly one-half of individuals with non-dialysis CKD and up to 60% of those receiving kidney replacement therapy [3]. Beyond its physical impact, persistent pain is associated with impaired mobility, sleep disturbance, depression, reduced quality of life and increased healthcare utilization [4]. Most research has approached this relationship from a single perspective: kidney disease leads to pain. Musculoskeletal disorders, peripheral neuropathy, CKD-mineral and bone disorder, vascular calcification, dialysis-related complications and multiple comorbidities all contribute to the high prevalence of pain in this population. As a result, the literature has largely focused on identifying safe analgesic strategies while avoiding drug-related nephrotoxicity [5,6].

Our understanding of chronic pain has evolved considerably over the past two decades. Chronic pain is no longer viewed just as ongoing nociceptive signaling but also as a complex disease characterized by maladaptive changes in the nervous, endocrine, immune and vascular systems [7,8]. Sustained sympathetic activation, hypothalamic–pituitary–adrenal axis dysregulation, chronic low-grade inflammation, oxidative stress, endothelial dysfunction and mitochondrial impairment all contribute to its pathophysiology. These same mechanisms are also recognized as major drivers of CKD progression, promoting inflammation, fibrosis, vascular injury and progressive nephron loss. Despite the remarkable overlap between the biology of chronic pain and the mechanisms driving CKD progression, little attention has been paid to the possibility that chronic pain itself may accelerate kidney function decline rather than simply accompany it [9,10].

The starting point for this hypothesis was the observation that chronic pain and CKD progression share multiple biological and clinical pathways that are usually considered separately. This prompted us to consider whether, when both conditions coexist for years, chronic pain may not simply reflect CKD severity but continuously reinforce mechanisms already driving renal injury. The question of whether effective pain control could influence CKD trajectory therefore emerged as a logical extension of this shared pathophysiology, rather than from evidence that analgesic treatment itself is renoprotective.

In this opinion, we argue that chronic pain should be considered not only a consequence of CKD but also a potential disease modifier capable of amplifying the biological pathways that drive progressive kidney injury.

## 2. Chronic Pain Is a Systemic Disease

### 2.1. Beyond Persistent Nociception: Chronic Pain as a Whole-Body Disorder

The traditional view of chronic pain as a prolonged symptom resulting from unresolved tissue injury has changed substantially over the past two decades. Rather than representing a simple extension of acute nociception, chronic pain is now recognized as a distinct disease characterized by chronic alterations in neural processing, maladaptive neuroplasticity and widespread biological changes that extend far beyond the original site of injury. In many patients, pain persists despite resolution of the initiating insult, while its intensity often correlates poorly with the degree of structural pathology, highlighting that mechanisms sustaining chronic pain become at least partially independent of the original trigger [11,12].

The nociceptive input induces functional and structural changes throughout the peripheral and central nervous systems, leading to amplification of pain signaling, impaired endogenous inhibitory pathways and abnormal sensory processing. These adaptations contribute not only to hyperalgesia and allodynia but also to widespread pain, fatigue, sleep disturbance, cognitive dysfunction and affective symptoms that frequently accompany chronic pain syndromes. Central sensitization should not be viewed solely as a pain mechanism but as a broader state capable of influencing multiple physiological systems [13,14].

Chronic pain is accompanied by reciprocal interactions between the nervous, endocrine, immune and vascular systems. Prolonged activation of neuroimmune pathways, glial cell signaling, autonomic imbalance and stress-related neuroendocrine responses establish a self-perpetuating cycle that maintains pain while simultaneously promoting systemic dysfunction. Consequently, chronic pain has increasingly been recognized as a multisystem disorder rather than an isolated sensory experience [12,15].

This broader perspective has important implications beyond pain medicine. Once chronic pain is understood as a sustained state of systemic dysregulation rather than a localized symptom, it becomes plausible that its consequences may extend to distant organs. Many of the pathways activated during chronic pain, including chronic inflammation, oxidative stress, endothelial dysfunction, autonomic dysregulation and mitochondrial impairment, are also recognized contributors to the progression of chronic kidney disease. This provides the rationale for considering whether chronic pain itself could actively influence the trajectory of CKD rather than simply coexist with it. The following sections examine the biological pathways that support this hypothesis and discuss how chronic pain may contribute to progressive kidney injury [16,17,18].

### 2.2. Neuroendocrine and Autonomic Dysregulation

One of the defining features of chronic pain is the prolonged activation of the body’s stress response systems. Repeated nociceptive input, together with the psychological burden of ongoing pain, continuously stimulates the hypothalamic–pituitary–adrenal (HPA) axis and the autonomic nervous system. While these responses are adaptive during acute injury, their continuous activation promotes widespread physiological changes that extend well beyond pain processing itself. Rather than restoring homeostasis, prolonged neuroendocrine activation establishes a maladaptive state characterized by hormonal imbalance and autonomic dysfunction [8].

Patients with chronic pain frequently exhibit alterations in cortisol secretion, impaired circadian regulation of glucocorticoid release and prolonged sympathetic activation accompanied by reduced parasympathetic tone. It contributes to increased resting heart rate, elevated vascular resistance, impaired baroreflex sensitivity and diminished heart rate variability, all of which reflect a permanent loss of physiological adaptability. At the same time, excessive catecholamine signaling influences immune cell activity, vascular function, and metabolic homeostasis, which creates conditions that favor stress rather than recovery [8,19].

Neuroendocrine and autonomic disturbances are also active contributors to chronic disease rather than secondary consequences of pain. Persistent stress signaling affects endothelial integrity, mitochondrial function, tissue perfusion and cellular metabolism while amplifying communication between the nervous, immune, and cardiovascular systems. They may initially represent compensatory mechanisms, but over time they become self-sustaining and contribute to progressive organ dysfunction. This provides a mechanistic framework through which chronic pain could influence the function of organs far removed from the primary site of nociception, including the kidneys [8,20,21].

## 3. Why Chronic Pain Could Accelerate CKD Progression

### 3.1. Sustained Inflammation and Immune Activation

Ongoing inflammation is widely recognized as one of the principal drivers of CKD progression. Regardless of the underlying etiology, immune activation promotes tubular epithelial injury, endothelial dysfunction, macrophage recruitment, fibroblast activation, and excessive extracellular matrix deposition, gradually shifting the kidney from adaptive repair toward irreversible fibrosis. So, rather than representing a secondary consequence of declining renal function, chronic inflammation actively contributes to disease progression throughout the course of CKD [22].

The hypothesis proposed in this Opinion raises an important question: could chronic pain sustain a systemic inflammatory environment that continuously reinforces these processes? Unlike acute pain, which elicits a transient inflammatory response that resolves with tissue healing, chronic pain is characterized by lasting neuroimmune activation that frequently outlasts the original injury. Activated glial cells, peripheral immune cells, and sensory neurons continuously release pro-inflammatory mediators, establishing a state of chronic low-grade inflammation that extends beyond the nervous system. Chronic pain is increasingly recognized as a systemic inflammatory condition rather than solely a disorder of chronic nociceptive signaling [23,24].

This inflammatory profile closely mirrors several pathways implicated in CKD progression. Continuous activation of NF-κB signaling, together with increased expression of cytokines like IL-1β, IL-6, and TNF-α, promotes leukocyte recruitment, endothelial activation and maladaptive tissue remodeling within the kidney. Chemokines and adhesion molecules further amplify inflammatory cell trafficking, while prolonged immune activation stimulates fibroblast proliferation and extracellular matrix accumulation, ultimately accelerating tubulointerstitial fibrosis and progressive nephron loss [25].

The importance of this overlap lies not in the presence of individual cytokines but in the possibility that two chronic conditions converge on the same inflammatory networks. Chronic pain is unlikely to initiate CKD through inflammation alone. However, by maintaining a systemic inflammatory background, it may continuously add to inflammatory processes already active within the diseased kidney, increasing the cumulative burden over time. This concept is consistent with the broader hypothesis that chronic pain acts as a disease modifier rather than a direct cause of kidney injury.

### 3.2. Oxidative Stress, Endothelial Dysfunction and Mitochondrial Injury

Oxidative stress is another fundamental mechanism driving CKD progression. Excessive production of reactive oxygen species (ROS) contributes to tubular epithelial injury, endothelial dysfunction, mitochondrial damage, and activation of profibrotic signaling pathways that ultimately promote irreversible nephron loss. Rather than representing a downstream consequence of kidney injury, oxidative stress actively interacts with inflammation, metabolic disturbances, and vascular dysfunction throughout the course of CKD, creating a self-perpetuating cycle of progressive renal damage [26].

An important question is whether chronic pain may further intensify this oxidative environment. Chronic nociceptive signaling is increasingly recognized as a source of ROS production through continuous activation of immune cells, mitochondrial dysfunction and redox-sensitive enzymatic systems [10]. Unlike the transient oxidative burst accompanying acute tissue injury, chronic pain is associated with prolonged systemic oxidative imbalance, reflected by increased circulating markers of lipid peroxidation, protein oxidation, and DNA damage [27]. These findings suggest that oxidative stress represents a systemic consequence of chronic pain rather than a process confined to the nervous system.

Oxidative stress has consequences that extend beyond direct cellular injury. Excess ROS rapidly reduce nitric oxide bioavailability through peroxynitrite formation, impair endothelial nitric oxide synthase activity, and promote endothelial activation. The resulting endothelial dysfunction increases vascular permeability, leukocyte adhesion, and microvascular injury while simultaneously amplifying inflammatory signaling. In this way, oxidative stress and endothelial dysfunction reinforce one another, establishing an environment that closely resembles the vascular abnormalities observed during CKD progression [19].

The kidney is particularly vulnerable to this cumulative oxidative burden because of its exceptionally high metabolic demand and dependence on mitochondrial ATP production, especially within proximal tubular epithelial cells [28]. Long-term ROS exposure disrupts mitochondrial respiration, damages mitochondrial DNA, and impairs oxidative phosphorylation, reducing cellular energy production while further increasing mitochondrial ROS generation. This cycle promotes tubular injury, inflammatory cell recruitment, fibroblast activation and extracellular matrix accumulation, all of which are well-established features of progressive CKD. Mitochondrial dysfunction is therefore at the center of kidney disease progression, rather than just a consequence of ongoing renal injury [28].

Chronic pain may not introduce a novel mechanism of kidney injury but instead continuously reinforce an interconnected network of oxidative, endothelial, and mitochondrial dysfunction already driving CKD progression. By increasing the cumulative oxidative burden over time, pain could strengthen biological processes that gradually shift the injured kidney toward irreversible functional decline.

### 3.3. Endothelial and Microvascular Dysfunction

Endothelial dysfunction has emerged as a central mechanism linking inflammation, oxidative stress, hypoxia, and fibrosis throughout CKD progression. Beyond serving as a passive vascular barrier, the endothelium actively regulates vascular tone, leukocyte trafficking, coagulation, permeability, and tissue perfusion. Progressive disruption of these functions contributes to microvascular injury, impaired oxygen delivery, and maladaptive tissue remodeling, making endothelial dysfunction an active driver of CKD progression rather than merely a consequence of declining kidney function [16].

So, could the vascular endothelium represent the interface through which chronic pain influences kidney disease? Chronic pain is increasingly recognized as a systemic condition characterized by chronic inflammation, oxidative stress and neuroendocrine activation, all of which directly affect endothelial function. Rather than acting independently, these mechanisms converge on the vascular endothelium, creating a persistent state of endothelial activation that extends far beyond the original site of nociception. The endothelium may therefore provide the missing link between chronic pain and progressive renal injury.

One of the earliest manifestations of endothelial injury is degradation of the endothelial glycocalyx, a carbohydrate-rich layer essential for maintaining vascular homeostasis. Loss of glycocalyx integrity increases vascular permeability, promotes leukocyte adhesion, reduces nitric oxide bioavailability, and facilitates endothelial activation long before irreversible structural vascular damage becomes apparent. Because chronic inflammation and oxidative stress are well-recognized consequences of pain, chronic pain may gradually promote systemic glycocalyx injury, establishing a vascular phenotype that favors widespread endothelial dysfunction even in the absence of overt cardiovascular disease [29].

These changes are particularly relevant to the kidney because the renal microcirculation depends on an intact endothelial network to preserve tissue oxygenation despite exceptionally high metabolic demands. Endothelial dysfunction within glomerular and peritubular capillaries impairs nitric oxide-mediated vasodilation, increases vascular resistance, disrupts autoregulatory responses, and reduces oxygen delivery to metabolically active tubular segments. Widespread tissue hypoxia subsequently activates hypoxia-inducible pathways, stimulates fibroblast activation, and promotes extracellular matrix deposition, mechanisms now recognized as major contributors to tubulointerstitial fibrosis and progressive nephron loss [23,30].

As CKD advances, endothelial injury further accelerates structural remodeling of the renal microvasculature. Progressive loss of peritubular capillaries limits oxygen diffusion throughout the tubulointerstitium, establishing a chronic hypoxic environment that perpetuates inflammation, oxidative stress, and fibrosis. At the same time, disrupted communication between endothelial cells, podocytes, tubular epithelial cells, mesangial cells, and pericytes compromises filtration barrier integrity and microvascular stability, further accelerating glomerulosclerosis and renal functional decline [31,32]. Viewed from this perspective, endothelial dysfunction is not simply another mechanism shared by chronic pain and CKD. It may represent the vascular pathway through which the systemic consequences of chronic pain are translated into progressive kidney injury.

### 3.4. Neuroendocrine and Autonomic Mechanisms

Kidney health depends on precise neurovascular regulation to maintain stable glomerular filtration despite continuous fluctuations in systemic blood pressure and metabolic demand. This balance is achieved through tightly coordinated interactions between the sympathetic nervous system, the HPA axis, and the renin–angiotensin–aldosterone system RAAS. Repeated disruption of these regulatory networks contributes to intraglomerular hypertension, impaired renal perfusion, chronic hypoxia, and progressive nephron loss, making neurohumoral dysregulation an important component of CKD progression [33,34].

This raises another important question: could chronic pain continuously disturb these homeostatic mechanisms? Persistent nociceptive input is one of the strongest chronic activators of the body’s stress response. Rather than producing a transient physiological reaction, ongoing pain maintains continuous sympathetic outflow, prolonged catecholamine release, reduced parasympathetic activity, impaired baroreflex sensitivity and diminished autonomic flexibility [35,36,37]. Over time, these adaptive responses become maladaptive, shifting cardiovascular regulation toward vasoconstriction and chronic neurohumoral activation.

The kidney is particularly susceptible to prolonged sympathetic stimulation because of its dense autonomic innervation. Increased renal sympathetic nerve activity promotes afferent and efferent arteriolar vasoconstriction, enhances tubular sodium reabsorption, and stimulates renin release from juxtaglomerular cells. This establishes a close interaction between the sympathetic nervous system and RAAS, with each pathway reinforcing the activity of the other. The resulting increase in intraglomerular pressure, sodium retention, and renal vascular resistance may gradually intensify mechanisms already contributing to CKD progression [38,39]. Neurohumoral disturbances interact closely with inflammatory, oxidative, and endothelial pathways. Sustained sympathetic activation reduces renal blood flow, compromises oxygen delivery to metabolically active tubular segments, and promotes chronic tissue hypoxia, while prolonged HPA axis dysregulation contributes to hypertension, insulin resistance, metabolic dysfunction, and immune imbalance. Rather than functioning as isolated systems, these pathways form an integrated stress response capable of amplifying multiple biological processes implicated in progressive kidney injury [40,41].

From this perspective, chronic pain may not simply increase sympathetic activity. Instead, it may chronically impair the physiological mechanisms that normally preserve renal homeostasis, exposing an already vulnerable kidney to repeated hemodynamic and neurohumoral stress.

### 3.5. Behavioral and Clinical Amplifiers

The mechanisms discussed in the previous sections are unlikely to fully explain how chronic pain could influence CKD progression. Chronic pain also alters everyday behaviors, treatment adherence, and long-term disease management, potentially increasing exposure to several established risk factors for kidney function decline [3,42]. Rather than acting independently, these clinical consequences may reinforce the pathways already driving CKD progression.

One of the earliest consequences of chronic pain is progressive loss of physical function. Patients often reduce physical activity because movement becomes painful, leading to deconditioning, sarcopenia, and prolonged sedentary behavior. Over time, these changes contribute to obesity, insulin resistance, hypertension, impaired cardiovascular fitness, and worsening metabolic health, all of which are recognized determinants of faster CKD progression. Sleep disturbance further compounds this burden by promoting sympathetic activation, impaired glucose metabolism, systemic inflammation, and reduced physiological recovery [43,44,45].

Chronic pain also changes the way patients engage with their disease. Depression, anxiety, fatigue, cognitive burden, and reduced motivation frequently accompany chronic pain and commonly coexist with CKD. These factors may substantially impair self-management by reducing adherence to antihypertensive and antidiabetic therapy, limiting participation in dietary and lifestyle interventions, and increasing missed clinic visits. Consequently, maintaining optimal blood pressure, glycemic control, and body weight becomes considerably more challenging despite their central importance for preserving kidney function. Pain may also influence therapeutic decision-making. Patients with uncontrolled symptoms are more likely to rely on frequent analgesic use, including nonsteroidal anti-inflammatory drugs despite their well-recognized nephrotoxicity. Even when nephrotoxic medications are successfully avoided, pain often limits participation in exercise programs, rehabilitation, and other non-pharmacological interventions that improve cardiovascular and metabolic health. As a result, pain management and kidney disease management become closely interconnected rather than separate components of care [46,47].

Unlike the molecular pathways discussed earlier, these behavioral and clinical amplifiers are potentially reversible. Effective pain management may restore physical activity, improve sleep quality, enhance treatment adherence, and facilitate healthier lifestyle behaviors [48]. Although it remains unknown whether these changes translate into slower CKD progression, they highlight an important concept: chronic pain may influence kidney disease not only through shared physiological mechanisms but also by shaping the clinical environment in which CKD is managed. Biological and behavioral effects support the hypothesis that chronic pain could function as a disease modifier rather than merely a symptom of chronic kidney disease.

## 4. A New Conceptual Framework: Chronic Pain as a Disease Modifier

Current CKD management largely considers chronic pain a consequence of declining kidney function rather than a factor capable of influencing disease progression [49]. Accordingly, pain assessment has traditionally focused on symptom severity, quality of life, functional impairment, and the safe use of analgesics, particularly with regard to nephrotoxicity [3,50]. Within this framework, successful pain treatment is regarded primarily as supportive care rather than an intervention capable of modifying the natural history of CKD.

The hypothesis proposed in this Opinion challenges that assumption. If the pain contributes to systemic dysregulation capable of reinforcing established mechanisms of kidney injury, should it still be viewed solely as a symptom of CKD? Or should it be considered another potentially modifiable factor influencing how rapidly kidney disease progresses? These questions represent more than a semantic distinction. They shift the clinical perspective from “How can pain be treated safely in patients with CKD?” to “Could effective pain control influence the natural trajectory of CKD itself?” While definitive evidence is currently missing, the overlap between the mechanisms sustaining chronic pain and those driving CKD progression provides a plausible rationale for investigating this possibility. Preliminary observational findings provide some support for investigating this hypothesis. In a retrospective cohort of more than 2.3 million US veterans with preserved baseline kidney function, moderate and severe pain were associated with a higher risk of rapid eGFR decline, with severe pain associated with a 17% higher adjusted odds of rapid kidney function decline [51]. Moderate and severe pain were also associated with approximately a 30% higher risk of the composite outcome of incident eGFR < 60 mL/min/1.73 m^2^ or death. However, findings are not uniform. In a prospective cohort of patients with pre-dialysis CKD, chronic musculoskeletal pain was independently associated with all-cause mortality but not with CKD progression or longitudinal eGFR decline. These studies provide us with an epidemiological signal [52]. If this hypothesis proves correct, chronic pain should no longer be regarded exclusively as a patient-reported outcome. Instead, it could become a prognostic variable incorporated into studies of CKD progression alongside established risk factors like hypertension, diabetes, albuminuria, and baseline kidney function. Future prospective cohorts should evaluate whether pain severity, duration, phenotype, or central sensitization independently predict kidney function decline after adjustment for conventional clinical variables. Likewise, mechanistic studies should determine whether successful pain management is accompanied by measurable changes in inflammatory activity, oxidative stress, endothelial dysfunction or other pathways implicated in CKD progression. Whether pain control modifies CKD progression would need to be tested in prospective interventional studies designed to minimize confounding by analgesic nephrotoxicity. A feasible approach would be to compare structured, kidney-safe pain management with usual care while avoiding or strictly controlling NSAID exposure in both groups. Serial assessment of pain severity, analgesic exposure, eGFR slope and albuminuria could then determine whether sustained pain reduction is associated with improved renal trajectories independently of nephrotoxic medication use. Non-pharmacological interventions could be particularly informative in this setting, as they may reduce pain without introducing direct pharmacological effects on kidney function [53,54].

An important alternative explanation should also be considered. Chronic pain may simply reflect more advanced CKD, greater comorbidity burden, or poorer overall health rather than independently influencing disease progression. Patients with more severe kidney disease frequently experience a higher prevalence of musculoskeletal disorders, neuropathy, frailty, depression, and reduced physical function, all of which may contribute to both greater pain and faster renal decline [55]. Any observed association between chronic pain and CKD progression does not necessarily imply a causal relationship. Also, the inflammatory, oxidative and vascular phenotype shared by chronic pain and CKD may also arise from common comorbidities rather than representing a direct effect of pain on the kidney. Diabetes and obesity, for example, can independently promote systemic inflammation, oxidative stress and endothelial dysfunction, while also being common among patients with CKD. Therefore, these overlapping pathways should not by themselves be interpreted as evidence of a causal pain–kidney relationship [56,57].

However, the consistent biological convergence between the mechanisms sustaining chronic pain and those driving kidney injury provides a positive rationale for investigating whether pain contributes to disease progression beyond serving as a marker of illness severity. Distinguishing these possibilities will require carefully designed longitudinal studies with appropriate adjustment for established clinical confounders.

A second question naturally follows. If chronic pain reinforces mechanisms that promote kidney injury, could pain management eventually become part of a kidney-protective strategy rather than solely supportive care? This does not imply that pain is a primary cause of CKD or that analgesic therapy is inherently renoprotective. Rather, it raises the possibility that reducing long-term nociceptive stress may lessen one component of the cumulative systemic burden acting on an already vulnerable kidney.

Similar conceptual shifts have occurred in other chronic diseases, where conditions once regarded as secondary complications were later recognized as modifiers of disease behavior through shared mechanisms rather than direct organ-specific injury [58,59,60,61]. Cardiovascular disease is a particularly relevant example, as chronic pain has been associated with cardiovascular morbidity and hypertension, with sustained sympathetic activation and systemic inflammation proposed as potential mediators [62]. A comparable bidirectional relationship has also been suggested between chronic pain and metabolic syndrome, where inflammatory pathways, physical inactivity and other pain-related behavioral changes may contribute to metabolic dysfunction [63]. These links raise a broader question of whether, in selected chronic diseases, persistent pain may participate in feedback loops that reinforce mechanisms underlying disease progression rather than functioning solely as a downstream symptom. Importantly, however, such associations do not establish that pain is a causal disease modifier or that pain control alters the natural history of these conditions and these relationships require prospective investigation. Whether chronic pain occupies a similar position in CKD remains unknown.

## 5. Clinical Implications

Although the hypothesis proposed in this Opinion remains to be confirmed, it highlights several potential implications for clinical practice. Current pain management in CKD primarily aims to relieve symptoms while minimizing treatment-related adverse effects, particularly nephrotoxicity. Declining kidney function also substantially affects the choice and dosing of therapies used for chronic pain. NSAIDs should generally be restricted and used cautiously because of their potential nephrotoxicity, while several commonly used analgesics and adjuvant agents require dose adjustment or extended dosing intervals because of reduced renal clearance. Particular caution is required with renally eliminated opioids and their active metabolites, as well as gabapentin and pregabalin, because accumulation may increase the risk of adverse effects. Pain management in CKD requires an individualized approach based on kidney function, drug pharmacokinetics and the risk–benefit profile of each intervention [46].

If chronic pain also contributes to biological processes associated with CKD progression, effective pain management may eventually prove relevant for reasons extending beyond symptom control alone.

This perspective also emphasizes the importance of comprehensive pain assessment in patients with CKD. Pain characteristics such as severity, duration, distribution and evidence of central sensitization may provide information that extends beyond quality of life and functional impairment. If future studies demonstrate independent associations between these features and kidney disease progression, pain assessment could become an additional component of CKD risk stratification alongside established clinical and laboratory markers. The proposed framework further supports a multidisciplinary approach to CKD management. Nephrologists, pain specialists, rehabilitation physicians, psychologists, physiotherapists and primary care physicians frequently address different aspects of chronic pain in isolation. However, if chronic pain contributes to mechanisms relevant to CKD progression, closer collaboration between these disciplines may become increasingly important. Integrating pharmacological and non-pharmacological pain management strategies with conventional kidney-protective therapies could represent a more comprehensive approach to patient care.

These considerations should not be interpreted as strong evidence that aggressive analgesic therapy slows CKD progression. Instead, the hypothesis presented here suggests that chronic pain deserves greater attention as a potentially modifiable systemic condition whose biological consequences may extend beyond symptom burden. Establishing whether effective pain control influences long-term renal outcomes is an important objective for future clinical research.

## 6. Conclusions

The relationship between chronic pain and CKD has traditionally been viewed as one-directional, with kidney disease leading to pain. This Opinion explores the possibility that the interaction may be more complex. Instead of acting solely as a consequence of CKD, pain may also influence the environment in which kidney disease progresses.

At present, this concept remains hypothetical and requires targeted prospective investigation. Future studies should determine whether pain severity independently predicts eGFR decline after accounting for comorbidities and nephrotoxic analgesic exposure, and whether sustained pain reduction is associated with slower CKD progression. Ultimately, prospective interventional studies using kidney-safe or non-pharmacological pain management, with longitudinal assessment of eGFR and albuminuria, will be needed to more directly test whether pain itself represents a modifiable component of CKD progression. By proposing this framework, our opinion may open the door to a new line of investigation into the pain–kidney relationship.

## Data Availability

No new data were created or analyzed in this study. Data sharing is not applicable to this article.
